# Tracking Progress Toward Elimination of Mother to Child Transmission of HIV in Zambia: Findings from the Early Infant Diagnosis of HIV Program (2009–2017)

**DOI:** 10.1093/tropej/fmz030

**Published:** 2019-05-14

**Authors:** Jane N Mutanga, Simon Mutembo, Amara E Ezeamama, Robert C Fubisha, Derrick Sialondwe, Brenda Simuchembu, Macwani Mutukwa, Jelita Chinyonga, Philip E Thuma, Christopher C Whalen

**Affiliations:** 1 Department of Pediatrics and Child Health, Livingstone Central Hospital, Livingstone, Zambia; 2 Department of Epidemiology and Biostatistics, College of Public Health, University of Georgia, Athens GA, 30602, USA; 3 Global Health Institute, College of Public Health University of Georgia, Athens GA, 30602, USA; 4 Southern Province Medical Office, Ministry of Health, Choma, 10101, Zambia; 5 Research Division, Department of Psychiatry, Michigan State University, East Lansing, MI, 48824, USA; 6 Macha Research Trust, Choma, 10101, Zambia

**Keywords:** HIV, early infant diagnosis, mother-to-child-transmission of HIV, risk factors, elimination, effectiveness of PMTCT

## Abstract

**Background:**

We carried out analyses of early infant testing results at Livingstone Central Hospital in Zambia to assess time of testing, linkages to care and availability of test results for clinical decision making.

**Methods:**

We abstracted data from registers of HIV-exposed infants who had dried blood spots cards (DBS) collected for DNA-PCR from January 2009 to December 2017. Only those tested from 2014 to 2017 had additional data which were used to estimate risk factors for mother-to-child HIV transmission using logistic regression models.

**Results:**

DBS were collected from 2630 children. The proportion of HIV-positive tests decreased from 21% in 2009 to 2% in 2016 and 2017. Median turnaround time for results was 9 weeks (IQR: 5, 15) for HIV-negative, 7 weeks (IQR: 5, 13) for HIV-positive children. Only 2% of infants whose mothers took antiretroviral therapy (ART) were HIV positive, while 18% of infants whose mothers took short course antiretroviral drugs (ARVs) were infected. Infants of mothers who did not take ARVs had 9 times the odds of an HIV positive test (OR = 8.9, 95% CI: 3.6, 22.6). Infants of mothers who received short course ARVs were 40% less likely to get an HIV test within the first 2 months of life (OR = 0.6, 95% CI: 0.4, 0.9) compared to infants of mothers who received ART. Only 52% had a third test at median age 52 weeks (IQR: 50, 54).

**Conclusions:**

Long turnaround time for test results and low retention in care after the initial HIV test were critical challenges to clinical decision making.

## INTRODUCTION

Global scale-up of Prevention of Mother-to-Child HIV Transmission (PMTCT) coverage led to 51% decline in new pediatric HIV infections among children from birth to 15 years of age since 2010 [1]. The major component of the HIV response for young children is effective PMTCT including primary HIV prevention for women in the reproductive age group and antiretroviral therapy (ART) for HIV-infected pregnant women.

In 2016, it was estimated that 83% of all pregnant women in Zambia were tested for HIV and 95% of those diagnosed with HIV, accessed ART [1, 2]. However, only 37% of HIV-exposed infants had an HIV DNA-PCR test done for HIV diagnosis within the first 2 months of life and only 46% of those with HIV positive tests accessed ART [2–4]. Early initiation of ART for HIV-infected infants is critical because it reduces the risk of mortality by as much as 76% [5]. Studies done in resource limited settings provided evidence that treatment of all HIV-infected pregnant women with ART (Option B+), followed by simplified prophylaxis and treatment algorithms for their infants resulted in low mother-to-child-transmission rates below 5% in breastfeeding populations [6, 7].

Early Infant Diagnosis (EID) is defined by the World Health Organization (WHO) as virologic testing of HIV-exposed infants by 2 months’ of age and is a key PMTCT intervention recommended by the WHO and adapted by the Zambian Ministry of Health [8, 9]. EID is challenging to implement in resource limited settings like Zambia because it requires the use of DNA-PCR which is only available at molecular laboratories in major cities [10, 11]. Maternal antibodies remain detectable in HIV exposed infant’s serum until 9–12 months of age, therefore, molecular techniques such as DNA-PCR are the definitive tests to detect HIV infection among infants. DNA-PCR gives reliable results and is effective at identifying infants infected in utero who have the highest risk of disease progression and mortality [8, 12, 13].

We carried out descriptive analysis on time of testing, linkages to care and availability of HIV test results for HIV-exposed infants at Livingstone Central Hospital (LCH), Zambia from January 2009 to December 2017. We discuss the challenges and successes of this PMTCT and pediatric HIV treatment program and generate program relevant information to inform institutional policy and/or investment in early infant diagnostic testing for HIV-exposed infants as a critical tool for ensuring AIDS free survival in the era of continued progress toward elimination of mother-to child-transmission of HIV in Zambia.

## METHODS

### Study design, site and population

We abstracted data from clinic registers and medical records of HIV-exposed infants and children at LCH in Livingstone, Zambia from January 2009 to December 2017. LCH is a tertiary level hospital that served a catchment population of over 1.2 million people in the Southern and parts of Western provinces of Zambia. This hospital served both an urban and rural population with an estimated HIV prevalence of 13.4% in 2016 [2, 14]. The only molecular laboratory that performed DNA-PCR since 2008 for the Southern part of Zambia was housed at LCH. The LCH had a pediatric center of excellence clinic that had been in operation since 2006 and had over 580 children on ART and close to 200 HIV-exposed infants in the EID program every year. The pediatric enter of excellence (PCOE) clinic offered treatment and care to HIV exposed and infected children in Livingstone district and surrounding areas. All HIV exposed infants who were born at LCH had dried blood spots cards (DBS) collected at birth from 2009. This was a pragmatic approach to diagnose HIV infection among exposed infants early and reduce mortality because early infant testing had been challenging especially in the surrounding rural areas. In addition, all hospitalized children were tested for HIV and this was documented to improve access to services for HIV prevention and care [15].

In our analysis, we included infants who were delivered at LCH and infants who were referred to the LCH from surrounding primary health care clinics following delivery complications like postpartum hemorrhage in the mother or birth asphyxia, birth defects or other complications of the newborn infant. We excluded very sick infants who were admitted to the neonatal intensive care unit, but included them if they survived beyond the first 28 days of life and were discharged from the neonatal intensive care unit and enrolled in care and follow-up at the PCOE clinic.

During the initial enrollment, demographic and clinical details of the tested mother–baby pair were entered into an exposed baby register and a medical record was opened for the exposed baby and kept at the PCOE clinic. The mother/caregiver was given the option to have the results sent to their usual primary health care clinic or to stay in care at the PCOE clinic.

Once the HIV DNA-PCR test results were received at the PCOE clinic, the caregiver was contacted by phone if a phone number was provided and asked to come back to the clinic to collect their results or go to their preferred primary health care clinic to collect the results. When the mother came back to the clinic, the result was communicated and a plan of care and further follow-up was initiated as follows: (1) The infant was scheduled to come back for repeat testing at 4–6 weeks of age, 6 months, 9 months, 12 months, 18 months of age and 3 months after cessation of breastfeeding, (2) prophylactic treatment with antiretroviral medications for a specified period of time, (3) Cotrimoxazole was prescribed if the baby was above 6 weeks of age.

All pregnant women who delivered at LCH received HIV counseling and testing following consent by the attending midwife. Dried blood spots cards were collected from all newborn infants whose mothers had confirmed HIV infection. Prophylactic ARV medications were prescribed for the infant and vaccines (Bacille Calmette Guerin (BCG) and oral polio vaccine (OPV)) are routinely administered before discharge at the hospital. However, if the infant required additional vaccines which are only administered at primary health facilities, follow-up visits were scheduled for the newborn at their primary health facility through the under five clinic. At the PCOE clinic, monthly clinical visits were scheduled for all the HIV exposed infants. At each clinical visit, the following parameters were assessed; (1) the child’s general health and wellbeing, (2) growth and neurodevelopment assessment, (3) nutritional status and infant feeding choice, (4) vaccine schedule, (5) HIV testing schedule, (6) prophylactic medication (ARVs and Cotrimoxazole), (7) Psychosocial assessment including living situation at home and contact details of the caregiver.

### Ethical considerations

This study was approved by the Zambia National Health Research Authority and the institutional review boards at Macha Research Trust and the University of Georgia. We conducted secondary analysis of anonymized routinely collected program data. Hence, informed consent was waived.

### Ascertainment of HIV exposure status

#### HIV testing for mothers

Pregnant women received HIV counseling and testing during antenatal clinic and upon admission to the delivery wards if their HIV status was unknown. Women who had HIV-negative test results during antenatal visits were retested for HIV as recommended by the Zambian Ministry of Health if the last test was more than six weeks before delivery [16]. HIV screening was done using a rapid test (Determine, Abbot Laboratories, USA) and all HIV positive results were confirmed using Unigold rapid test (TM HIV, Trinity Biotech, Ireland).

#### HIV testing for infants

Trained nurse counselors collected whole blood from infants of HIV-infected mothers using heel or big toe prick and the blood was spotted onto 5 circles on filter paper. The filter paper was dried overnight at room temperature and sealed in humidity free bags and later sent to the molecular laboratory. One spot from each filter paper was tested by COBAS Ampliprep/Taqman HIV-1 Version 2.0 real time PCR assay (ROCHE Diagnostics, Indianapolis, IN) according to manufacturer instructions. The date of collection of the dried blood spots card and subsequent result and prophylaxis medication was recorded in the child’s medical record. All positive tests were confirmed by collecting a second sample from the child and performing another DNA-PCR test. However, medication was commenced without the confirmatory test. All the children at the PCOE clinic received confirmatory tests.

#### Mother–baby pair follow-up

The duration of follow-up was from time of delivery until 3 months after cessation of breastfeeding or 2 years of age, for children who stopped breastfeeding earlier. Dried blood spots cards for HIV DNA-PCR testing were collected at 6 weeks and 6 months of age. Routine rapid HIV antibody tests were done for children above 12 months of age. If the test result was positive for HIV antibodies, DNA-PCR was done to confirm HIV infection. HIV-infected children were immediately commenced on ART as recommended by the Ministry of Health HIV treatment guidelines [16]. Birth testing of all HIV-exposed infants was implemented at LCH in 2013. The final outcome test was done with a rapid HIV test at least 3 months after cessation of breastfeeding for children aged above 18 months who continued breastfeeding and at 18 months of age for children who stopped breastfeeding earlier. If the rapid HIV test was positive, DBS was collected for HIV DNA-PCR.

During the follow-up period, the infants were scheduled to come back to the clinic at least once a month for routine growth and neurodevelopment assessments. At the initial enrollment session, the caregivers and counselors discussed the infant testing schedule, how to collect the DNA-PCR test results and how to access care for the child among other things. The counselors obtained consent to make phone calls and/or home visits if the child missed their scheduled appointments. The DNA-PCR test results were usually given to the caregivers at the next scheduled appointment. Those who missed their scheduled appointment were contacted by phone or home visits and encouraged to come for the clinic appointment and receive their result. Clients with HIV-positive test results were followed up more frequently by the clinic outreach team to ensure that they came back to receive the test results and access ART. The outreach team facilitated linkage to care for families who opted to start treatment at a primary health facility near their home. The outreach team was comprised of a nurse counselor, a social worker and some community volunteers.

### Data collection

We abstracted routinely collected clinical data from HIV-exposed baby registers. The HIV-exposed baby registers contain baseline demographics, delivery and HIV test history of all the infants born to HIV-infected women at LCH. We abstracted the following variables: file number, date of birth, gender, address, date of collection of the DBS, and the result of the HIV DNA-PCR test. After implementation of Option B+ in 2013, the registers were updated to include the following data: date of collection of first DBS, test result, date of collection of second DBS and respective test result, date of collection of third test and the respective test result. In addition, we abstracted the mother’s details as follows: when did the mother know her HIV status, when was the mother commenced on ART, what regimen of ARVs did she take, did the baby take ARVs for prophylaxis, and what was the infant feeding choice. We traced the individual patient records through the clinic registry and utilized them to complete the records from the exposed baby registers.

### Statistical analysis

We performed descriptive analysis of the baseline data from the mother–baby pairs by calculating medians, interquartile ranges, frequencies, and percentages by HIV infection status as confirmed by the DNA-PCR test results recorded in the HIV-exposed baby registers. We present descriptive statistics for categorical variables and medians with interquartile range (IQR) for continuous variables. Univariate logistic regression evaluated the infant’s HIV test (positive vs. negative) and timing of the infant’s HIV testing, (early before 2 months of age vs late after 2 months of age) and how this was affected by the following risk factors; (1) timing of the mother’s first HIV diagnosis, (2) whether or not the mother received ARVs during pregnancy and, (3) whether the infant received ARVs for prophylaxis during the first 6 weeks of life. Data analysis were performed using SAS software (SAS version 9.4 (SAS Institute Inc, Cary, NC).

## Results

**Fig. 1.**
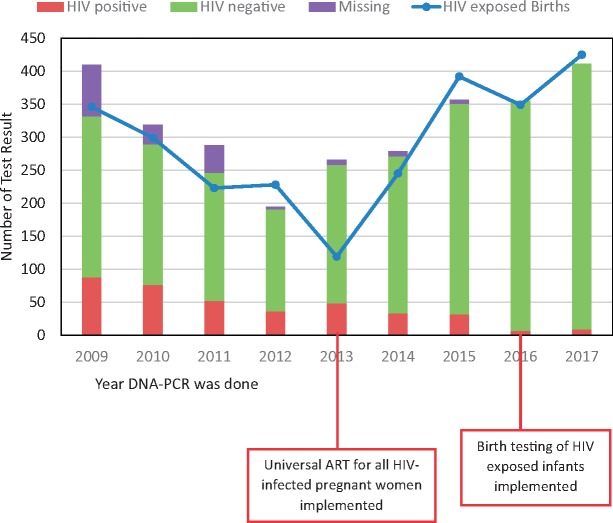
Early Infant diagnostic test results for HIV diagnosis for children at LCH: 2009–2017. *includes all DNA-PCR tests done at the hospital including in the delivery ward and NICU. Some of the patients were not enrolled in the HIV clinic at the hospital and the result was sent to their primary healthcare facility, hence not included in Table 1.

Dried blood spots cards were collected from 2680 children for HIV DNA-PCR at LCH between January 2009 and December 2017. Records from the maternity wards indicated that in 2017, at least 98% of all women who delivered at LCH had received HIV testing and 99% were retested at the time of delivery and 99% of the infants born at LCH were tested for HIV at birth. The number of HIV-exposed deliveries is different from the number of tested children because some of those tested were not delivered at LCH but were referred from primary health care facilities (Fig. 1). Among the referred patients, 89% were admitted to the pediatric inpatients wards and treated for medical conditions including pneumonia, diarrhea and neonatal sepsis, at least 3% were self-referrals who already had another child in care at the clinic and 8% were referrals for treatment of medical conditions such as congenital heart disease, birth defects or delayed milestones. At the start of the testing program in 2009, 19% (*n* = 78/410) of the tested children did not receive their results, but by 2016 all the tested children received their results (Fig. 1). The proportion of HIV-positive tests decreased from 21% (*n* = 88/410) in 2009, to 2% in 2016 (*n* = 7/355) and 2% in 2017 (*n* = 9/411) (Fig. 1).

Of total 2680 tested children, 659 came to the clinic during the post Option B+ era (2014–2017) after the registers and medical records had been updated and baseline demographic data for these children were available for analysis (Table 1). We were able to analyze demographic data for children who were attended to after implementation of Option B+. All the children were tested at first contact with the hospital staff usually after delivery, during hospital admission or at first contact with the out patient’s clinic following referral from primary health care. We found that the median age of the children at the time of the 1st DNA-PCR test was 3 weeks (IQR: 0, 9) for children who had an HIV-negative test results and 7.5 weeks (IQR: 2, 28) for children with HIV-positive test result. The median turnaround time for results to get back to the caregivers was 9 weeks (IQR: 5, 15) for HIV-negative children and 7 weeks (IQR: 5, 13) for HIV-positive children (Table 1).

**Table 1:** Baseline characteristics of infant’s first DNA-PCR for HIV diagnosis at LCH: 2014–2017

Characteristic	Result of baby's first DNA-PCR test		
	Negative	Positive	Total
	*N* = 579	*N* = 80	*N* = 659
**Age in (weeks) when 1st DNA-PCR was done (Median IQR)**	3 (0–9)	7.5 (2–28)	
**Time (weeks) from collection of DBS to result given to caregiver Median (IQR)**	9 (5–15)	7 (5–13)	
**Sex *N* (%)**			
Female	285 (89)	35 (11)	320
Male	290 (87)	44 (13)	334
Missing	4 (80)	1 (20)	5
**Time of mother’s first HIV diagnosis *N* (%)**			
Before pregnancy	46 (94)	3 (6)	49
During pregnancy	453 (89)	55 (11)	508
During labor and delivery or PNC	38 (83)	8 (17)	46
Unknown	42 (75)	14 (25)	56
**Mother received ARVs for PMTCT *N* (%)**			
NVP or AZT only during ANC or delivery	316 (82)	71 (18)	387
ART	200 (98)	5 (2)	205
none	63 (94)	4 (6)	67
**Baby received ARVs for prophylaxis before 6 weeks of age *N* (%)**			
Yes	452 (89)	56 (11)	508
No	127 (84)	24 (16)	151
**Year DNA-PCR was done *N* (%)**			
2014	146 (82)	33 (18)	179
2015	119 (79)	31 (21)	150
2016	167 (96)	7 (4)	174
2017	147 (94)	9 (6)	156

PNC, prenatal clinic; ART, antiretroviral therapy; NVP, nevirapine; AZT, azido thymidine; PMTCT, prevention of mother to child transmission of HIV; DBS, dry blood spot; IQR, interquartile range. 98% of the infants in this population were breastfed. 96% of HIV infected infants were linked to care and treatment. Only children with demographic information recorded in the exposed baby registers at the pediatric HIV clinic (PCOE) are included in this table.

At least 8% of the mothers did not know their HIV infection status during pregnancy and were not tested during delivery. Of these mothers, we found that 25% (*n* = 14/56) of their infants were diagnosed with HIV. A total of 7% (*n* = 46/659) mothers tested positive for HIV during delivery or during the first 6 weeks after delivery and 17% (*n* = 8/46) of their infants were HIV-infected. Among infants of mothers who were diagnosed with HIV during pregnancy, 11% (*n* = 55/508) were diagnosed with HIV and among mothers who knew their HIV infection status before pregnancy, only 6% (*n* = 3/49) were diagnosed with HIV.

From the logistic regression model, infants of mothers who did not know their HIV infection status during pregnancy, delivery and postnatal had five times the odds of an HIV positive diagnosis (OR = 5.1, 95% CI 1.4, 19.0) (Table 2). Only 2% (*n* = 5/205) infants whose mothers took ART during pregnancy tested positive for HIV while 18% (*n* = 71/387) infants whose mothers took short course antiretroviral drugs (Nevirapine (NVP) or Zidovudine (AZT)) during pregnancy or delivery were diagnosed with HIV infection and 6% (*n* = 4/67) infants of HIV infected mothers who did not take any ART were diagnosed with HIV (Table 1). Infants of HIV-infected mothers who did not take any ART during pregnancy had 9 times the odds of an HIV positive diagnosis (OR = 8.9, 95% CI: 3.6, 22.6) (Table 2).

**Table 2:** Maternal factors associated with likelihood of positive infant PCR test and early infant testing

	Result of infant’s PCR test (positive vs. negative)			Early testing (before 2 months of age) vs. late testing		
	OR	95% CI	*p*	OR	95% CI	*p*
**Time of mother’s first HIV diagnosis**						
Before pregnancy	ref			ref		
During pregnancy	1.9	0.6, 6.2	0.3105	0.9	0.5, 1.8	0.9526
During labor or postnatal	3.2	0.8, 13.2	0.0996	1.9	0.8, 5.1	0.1512
Unknown	**5.1**	**1.4, 19.0**	**0.0150**	1.9	0.8, 4.8	0.1313
**Mother received ARVs for PMTCT**						
ART	ref			ref		
Monotherapy (NVP or AZT during ANC)	2.5	0.7, 9.7	0.1745	**0.6**	**0.4, 0.9**	**0.0092**
None	**8.9**	**3.6, 22.6**	**<.0001**	1.05	0.5, 2.0	0.8695
**Baby received ARVs for prophylaxis before 6 weeks**						
Yes	ref			ref		
No	1.2	0.9, 1.3	0.1096	**0.9**	**0.8, 0.9**	**0.0404**

NVP, Nevirapine; AZT, Azidothymidine; ANC, antenatal clinic. Bolded numbers were statistically significant.

### Timing of first PCR HIV test

In 2016, 45% (*n* = 78/174) of infants were tested within the first 6 days of life (birth testing) which was a marked increase from 2014 when only 32% (*n* = 58/179) of the infants tested during the same time period. In 2017, 78% (*n* = 7/9) of the HIV-infected infants were diagnosed with tests obtained during the first week of life and in 2014, 15% (*n* = 5/33) of the HIV infections were diagnosed from tests obtained during the first week of life (Table 3).

**Table 3:** Early infant testing with DNA-PCR for HIV diagnosis by age group at Livingstone Central Hospital: 2014–2017

Year	Total tested at birth	Birth to 6 days	Total tested at age 1–4 weeks	1–4 weeks	Total tested at age 4->8 weeks	4–8 weeks	Total HIV positive	Total tested *N*
	*N* (%)	Positive	*N* (%)	Positive	*N* (%)	Positive		
		*N*		*N*		*N*	*N*	
2014	58 (32)	5	38 (22)	3	83 (46)	25	33	179
2015	28 (19)	1	31 (20)	7	91 (61)	23	31	150
2016	78 (45)	2	38 (23)	2	58 (32)	3	7	174
2017	67 (43)	7	25 (16)	1	64 (41)	1	9	156

Option B+ was implemented in 2013 at Ministry of Health facilities in Zambia. Birth testing was implemented in 2016.

Infants of mothers who received monotherapy antiretroviral drugs were 40% less likely to get an HIV test within the first 2 months of life (OR = 0.6, 95% CI: 0.4, 0.9). Infants who did not take ARVs for prophylaxis during the first 6 weeks of life were 20% less likely to get tested within the first 2 months of life (OR = 0.8, 95% CI: 0.7, 0.9) (Table 2).

### Linkage to care

We found that 98% (*n* = 646/659) infants were breastfed and 78% (*n* = 508/649) took Nevirapine for post exposure prophylaxis during their first 6 weeks of life. All the infants who were prescribed ARVs for post exposure prophylaxis were documented to have completed treatment up to at least 6 weeks or 6 months of age for infants whose mothers did not receive ARVs during pregnancy. Overall, 96% (*n* = 77/80) of infants diagnosed with HIV infection were commenced on ART within a median of 3 days (IQR: 0, 4) after the caregiver received the result. The registers recorded several phone calls and home visits and counseling sessions for at least 25% (*n* = 20/80) of HIV-positive infants before they were successfully linked to treatment. Some of the phone calls and home visits were made before the caregiver came to collect the result and some were after the results were given to the caregiver. Of the three infants who didn’t commence ART, one died during the second week of life due to neonatal complications, one infant’s caregiver refused to have the baby commenced on ART and one child was lost to follow-up.

### Subsequent HIV testing

Of the 659 infants and children who had HIV DNA-PCR tests, 71% (*n* = 469) had the scheduled second subsequent HIV test done at median age of 24 weeks (IQR; 24, 28). Of those who got a second subsequent HIV test only 1% (n = 6) were HIV-positive. A total of 52% (*n* = 344) had a third subsequent HIV test at median age of 52 weeks (IQR: 50, 54) and of these only 0.6% (*n* = 2) were diagnosed with HIV infection (Table 4). For 12% (*n* = 23/190) of the medical charts of children who did not come back for their second subsequent HIV test, it was recorded that the family had requested to be referred to a health facility near their home for follow-up care.

**Table 4:** Children who came back for subsequent HIV tests at Livingstone Central hospital: 2014–2017

	*N*	Age (weeks) median (IQR)	Negative tests *N* (%)	Positive tests *N* (%)
1st Test	659	3 (0–11)	579 (89%)	80 (12%)
2nd Test	469	24 (24–28)	463 (99%)	6 (1%)
3rd Test	344	52 (50–54)	342 (99.4)	2 (0.6%)

IQR, interquartile range.

## Discussion

In this study, we observed that availability of HIV-DNA test results improved and all the infants whose samples were collected in 2016 and 2017 received their test results. After implementation of Option B+ in 2012, the documentation of maternal clinical history and clinical follow-up parameters of HIV-exposed children improved over time and by 2014, we were able to evaluate important PMTCT outcomes such as age of testing, turnaround time of results and maternal HIV diagnosis and treatment history.

Birth testing was implemented in Zambia in 2015–2016 but all HIV-exposed infants born at this health facility were tested soon after birth as a pragmatic approach to improve early infant testing especially among families from rural areas who were unlikely to access timely HIV testing for their infants. However, we observed an increase in uptake of birth testing after Option B+ implementation in 2012–2014.

Our finding that Infants of HIV-infected mothers who did not take any ART during pregnancy had 9 times the odds of an HIV positive diagnosis when compared to infants of mothers on ART demonstrates that infants of mothers who did not take ART have much higher risk of an HIV positive diagnosis. We observed decline in HIV positivity from 21% (*n* = 88/410) in 2009, to 2% in 2016 (*n* = 7/355) and 2% in 2017 (*n* = 9/411). Our findings are consistent with reports by the Centers for Disease Control and prevention that the HIV DNA-PCR positivity at national level in Zambia declined to 2% in 2015 [17]. A prospective cohort study done in peri-urban settings in Zambia reported infant HIV free survival of 99.0% (95% CI: 98.0–99.5%) at 6 weeks, 97.5% (95% CI: 96.1–98.4%) at 6 months and 96.3% (95%CI: 94.8–97.4%) at 12 months [18]. They reported that loss to follow-up was only 5% at later testing points. In contrast, retention in care was a critical challenge in our study as demonstrated by our findings that 71% infants came back for their second test and only 52% came back for a third test. Retention in care needs to be strengthened so that outcomes of HIV exposed infants can be clearly understood and their needs can be met.

The clinic outreach team made several attempts to engage caregivers of children who missed appointments but retention in care was still low especially among children with HIV negative tests. One reason for the low subsequent HIV testing is that 12% of the clients decided to seek services from primary health care facilities near their homes as recorded in the medical charts but we could not establish reasons for the majority of the clients. A study carried out in South Africa documented low levels of retesting and retention in care especially among HIV-exposed infants with HIV-negative test results [13]. Similarly, this study did not establish the reasons for low subsequent testing.

Another critical challenge observed in this program was the long turnaround time for results to get back to the caregivers. This resulted in delayed clinical decision making for HIV-infected infants and children. This critical finding is not unique to our analysis but has been reported by other studies. A study from a rural setting in Zambia, showed that median turnaround time for results to get back to the caregivers was 13 weeks (IQR: 7, 21), which was longer than what we found [19]. The longest delay in this study was the interval from the time the results arrived at the clinic to the time the result was given to the caregiver and this was attributed to delays in processing and communicating the results to the caregivers [19]. In a study from Lesotho, the mean turnaround time was 8 weeks [20]. In our study, the HIV tests were done within the same health facility and we expected the turnaround time to be shorter. The major reasons for the long turnaround times at this health facility was long service times when the PCR machine required maintenance. We observed three periods of time when the laboratory machines had stopped working and the machine service process went on for more than 2 months. These three periods of time increased the median turnaround time and clients seen during these periods had at least two scheduled visits before they could get their results.

HIV-infected children in this cohort were tested at older median age and this represents differential delay in the timing of HIV testing and has critical clinical implications because HIV-positive children who are a priority for early initiation of ART were actually delayed.

A major strength of this program at LCH is that there was staff dedicated to the counseling, testing, treatment and follow-up of HIV-exposed and HIV-infected children and their caregivers. The presence of staff dedicated to outreach made it possible to track clients and 96% of those in need of treatment had access in a timely manner. Although results indicate that loss to follow-up was still high especially among those with HIV negative test results, the outreach team played a very important role in the success of the program.

A limitation of our study is that we analyzed routinely collected program data and had inconsistencies and missing information, but we resolved this by collecting additional data from the patient medical charts. We found that the registers were useful in helping us understand the pediatric antiretroviral treatment program, specifically the uptake of early infant testing at LCH. Our findings were further limited by the low subsequent testing as only 52% of the initial tested children came back for their 3rd subsequent test and we could not account for the children who were lost to follow-up. Another limitation is that we used early infant testing results from one health facility and cannot infer our results to national level. However, the findings from this health facility enabled better understanding of the successes and challenges of the early infant testing program at facility and this will potentially help to inform institutional policy and will be useful in similar settings.

## Conclusion

Long turnaround time for HIV DNA-PCR test results to get back to the patients and low retention in care after the initial HIV test was a challenge to clinical decision making. Documentation of patient information, processing and communication of test results to the care givers needs to be improved to facilitate monitoring of patient outcomes like HIV free child survival which are critical to inform policy. We recommend to program managers and clinicians to develop effective interventions to improve retention in care and strengthen early infant HIV testing.

## References

[1] Joint United Nations Programme on HIV/AIDS U. Fact Sheet: Global HIV & AIDS Statictics - 2018 http://www.unaids.org/en/resources/fact-sheet (11 December 2018, date last accessed).

[2] Pepfar Z. Zambia Country Operational Plan (COP) 2016 Strategic Direction Summary. Lusaka, Zambia: US dept of State - Presidents' Emergency fund for AIDS Relief (PEPFAR); 2016.

[3] Ministry of Health Z, National AIDS Council, Zambia. Zambia Country Report: Monitoring the Declaration of Commitment on HIV/AIDS and the Universal Access. Lusaka: Zambia. 2015.

[4] Pepfar Z. Zambia Country Operational Plan (COP) 2017, Strategic Direction Summary. Lusaka, Zambia: US Dept of State–Presidents' Emergency Fund for AIDS Relief (PEPFAR); 2017.

[5] Violari A, Cotton M, Gibb D, et al Early antiretroviral therapy and mortality among HIV-infected infants. N Engl J Med 2008;359:2233–44. 19020325 10.1056/NEJMoa0800971 PMC2950021

[6] Kim MH, Ahmed S, Preidis GA, et al Low rates of mother-to-child HIV transmission in a routine programmatic setting in Lilongwe, Malawi. PLoS One 2013;8:e64979. 23741437 10.1371/journal.pone.0064979 PMC3669205

[7] Hsiao N-Y, Stinson K, Myer L. Linkage of HIV-infected infants from diagnosis to antiretroviral therapy services across the Western Cape, South Africa. PLoS One 2013;8:e55308. 23405133 10.1371/journal.pone.0055308 PMC3566187

[8] WHO. Consolidated Guidelines on the Use of Antiretroviral Drugs for Treating and Preventing HIV Infections: Recommendations for a Public Health Approach. Geneva, Switzerland: World Health Organisation; 2016. 27466667

[9] Ministry of Health Z. Zambia Consolidated Guidelines for Prevention and Treatment of HIV Infection. Lusaka, Zambia: Ministry of Health, Zambia; 2018.

[10] Dunn DT, Brandt CD, Krivine A, et al The sensitivity of HIV-1 DNA polymerase chain reaction in the neonatal period and the relative contributions of intra-uterine and intra-partum transmission. AIDS 1995;9:F7–11. 8527070 10.1097/00002030-199509000-00001

[11] Comeau AM, Pitt J, Hillyer GV, et al Early detection of human immunodeficiency virus on dried blood spot specimens: sensitivity across serial specimens. J Pediatr 1996;129:111–8. 8757570 10.1016/s0022-3476(96)70197-x

[12] Celletti F, Sherman G, Mazanderani A. Early infant diagnosis of HIV: review of current and innovative practices. Curr Opin HIV AIDS 2017;12:112–6. 27941493 10.1097/COH.0000000000000343

[13] Technau K-G, Kuhn L, Coovadia A, et al Improving early identification of HIV-infected neonates with birth PCR testing in a large urban hospital in Johannesburg, South Africa: successes and challenges. J Int AIDS Soc 2017;20:1–8. 10.7448/IAS.20.01/21436 PMC5515050 28406596

[14] MOH/CSO/PEPFAR/ICAP/CDC/TDRC/UTH/UNZA. Zambia Population-Based HIV Impact Assessment ZAMPHIA 2015-2016. 2016.

[15] Mutanga J, Raymond J, Towle M, et al Institutionalizing provider-initiated HIV testing and counselling for children: an observational case study from Zambia. PLoS One 2012;7:e29656. 22536311 10.1371/journal.pone.0029656 PMC3335043

[16] Ministry of Health Z. Zambia Consolidated Guidelines for Treatment and Prevention HIV Infection. Lusaka, Zambia: Ministry of Health; 2016.

[17] Diallo K, Kim AA, Lecher S, et al Early diagnosis of HIV infection in infants - one Caribbean and six Sub-Saharan African countries, 2011-2015. MMWR Morb Mortal Wkly Rep 2016;65:1285–90. 27880749 10.15585/mmwr.mm6546a2

[18] Chi BH, Mutale W, Winston J, et al Infant HIV-free survival in the era of universal antiretroviral therapy for pregnant and breastfeeding women: a community-based cohort study from rural Zambia. Pediatr Infect Dis J 2018;37:1137–1141. 29601456 10.1097/INF.0000000000001997 PMC6160366

[19] Sutcliffe C, van Dijk J, Hamangaba F, et al Turnaround time for early infant HIV diagnosis in rural Zambia: a chart review. PLoS One 2014;9:e87028. 24475214 10.1371/journal.pone.0087028 PMC3901716

[20] Tiam A, Gill MM, Hoffman HJ, et al Conventional early infant diagnosis in Lesotho from specimen collection to results usage to manage patients: where are the bottlenecks? PLoS One 2017;12:e0184769. 29016634 10.1371/journal.pone.0184769 PMC5634554

